# MicroRNA: An Emerging Predictive, Diagnostic, Prognostic and Therapeutic Strategy in Ischaemic Stroke

**DOI:** 10.1007/s10571-020-01028-5

**Published:** 2020-12-24

**Authors:** Rais Reskiawan A. Kadir, Mansour Alwjwaj, Ulvi Bayraktutan

**Affiliations:** Stroke, Division of Clinical Neuroscience, School of Medicine, The University of Nottingham, Clinical Sciences Building, Hucknall Road, Nottingham, NG5 1PB UK

**Keywords:** microRNA, Stroke, Biomarkers, Therapy, Diagnostic marker, Prognostic marker

## Abstract

Stroke continues to be the third-leading cause of death and disability worldwide. The limited availability of diagnostic tools approved therapeutics and biomarkers that help monitor disease progression or predict future events remain as the major challenges in the field of stroke medicine. Hence, attempts to discover safe and efficacious therapeutics and reliable biomarkers are of paramount importance. MicroRNAs (miRNAs) are a class of non-coding RNAs that play important roles in regulating gene expression. Since miRNAs also play important roles in key mechanisms associated with the pathogenesis of stroke, including energy failure, inflammation and cell death, it is possible that miRNAs may serve as reliable blood-based markers for risk prediction, diagnosis and prognosis of ischaemic stroke. Discovery of better neurological outcome and smaller cerebral infarcts in animal models of ischaemic stroke treated with miRNA agomirs or antagomirs indicate that miRNAs may also play a cerebrovascular protective role after an ischaemic stroke. Nonetheless, further evidences on the optimum time for treatment and route of administration are required before effective translation of these findings into clinical practice. Bearing these in mind, this paper reviews the current literature discussing the involvement of miRNAs in major pathologies associated with ischaemic stroke and evaluates their value as reliable biomarkers and therapeutics for ischaemic stroke.

## Introduction

Stroke is characterised by an acute neurological dysfunction lasting for more than 24 h or leading to death with no apparent cause other than vascular origin. There are two major types of stroke depending on aetiology: ischaemic and haemorrhagic. Ischaemic strokes stem from the occlusion of the blood vessel leading to or within the brain due to formation of a thrombus (thrombotic strokes) or an embolus (embolic strokes). Haemorrhagic strokes, on the other hand, result from the rupture of an artery within (intracerebral haemorrhage) or on the surface (subarachnoid haemorrhage) of the brain. Ischaemic strokes account for approximately 85% of all strokes and therefore represent the main subtype of stroke (Benjamin Emelia et al. 2019; Johnson et al. 2019).

Due to high rate of mortality, morbidity and socio-economic costs for patients, families and society in general, stroke constitutes one of the major healthcare issues in the world (Johnson et al. 2019; WHO 2019). Considering that the majority of strokes are preventable, an appropriate risk assessment and integrated strategy for stratification of the general population via predictive biomarkers may be the best option to reduce the number of future strokes in individuals who are classified at high risk (Powers 2020). The diagnostic modality for stroke cannot merely rely on neuroimaging techniques such as computed tomography (CT) and magnetic resonance imaging (MRI) which are expensive, somewhat ineffective to recognise early signs of infarct and unavailable in most hospitals in low-income countries (Parody et al. 2015; Wardlaw Joanna et al. 2007). Similarly, therapeutic strategy cannot solely rely on recombinant tissue plasminogen activator (rtPA) and mechanical thrombectomy, as such strategies are expensive, fraught with high risk of haemorrhagic complications and require highly trained personnel (Kadir and Bayraktutan 2020). In addition, the unavailability of prognostic biomarkers for stroke has to be addressed, so that unfavourable outcomes, notably haemorrhagic transformation and stroke recurrence, can be anticipated at an early stage (Faraji et al. 2013; van Kranendonk Katinka et al. 2019). Taken together, point-of-care diagnostic tool, reliable prognostic marker as well as low-cost and widely accessible treatment strategy are urgently needed to prevent people from getting stroke and also to improve stroke care and interventions once they have had a stroke.

Accumulating evidence has shown the existence of an intricate relationship between microRNAs (miRNAs) and the major mechanisms, including energy failure, excitotoxicity, oxidative stress, inflammation, cell death and blood–brain barrier (BBB) disruption, implicated in the pathogenesis of ischaemic stroke (Li et al. 2013). As a consequence, miRNAs have attracted a great deal of attention as potential blood-based biomarkers to predict, diagnose and evaluate the prognosis of ischaemic stroke and also as therapeutics to treat ischaemic stroke (Vijayan and Reddy 2016a; Xu et al. 2018). Such comprehensive approach, ranging from an accurate prediction of healthy individuals who may suffer an ischaemic stroke in the future to an efficacious treatment of ischaemic stroke, is urgently required to effectively address the deteriorating impact of stroke.

## Biogenesis of miRNA

miRNAs are small non-coding RNAs that consist of approximately 20–22 nucleotides and play a crucial role in the post-transcriptional regulation of gene expression. The majority of miRNAs are initially transcribed from genomic DNA by RNA polymerase II to generate primary miRNA (pri-miRNA). Pri-miRNA consists of long nucleotide chains, hundreds or thousands of base pairs in length and containing at least one hairpin loop (Treiber et al. 2019). In general, biogenesis of miRNA includes a canonical and a non-canonical pathway. In canonical pathway, pri-miRNA is recognised by the nuclear enzyme DiGeorge Syndrome Critical Region 8 (DGCR8) and cleaved by RNAase III endonuclease Drosha, which in turn forms precursor miRNA (pre-miRNA). Subsequently, exportin-5 translocates pre-miRNA from the nucleus into cytoplasm and forms 22-nucleotide double-stranded RNA, containing the mature miRNA guide strand and the passenger (miRNA*) strand through a cleavage process by another RNAase III endonuclease enzyme, Dicer (Kumar and Reddy 2016). In contrast, in non-canonical pathway, miRNA biogenesis is performed through Drosha- or Dicer-independent pathways. The notable class of Drosha-independent pathways are the mirtrons which originate from introns that function as pre-miRNAs once spliced and hence do not require cleavage by Drosha and are directly exported to the cytoplasm for processing by Dicer (Ruby et al. 2007). Conversely, pre-miRNA in Dicer-independent pathway is produced by Drosha, without bypassing the cleavage process by Dicer and immediately exported to the cytoplasm (Yang et al. 2010). Ultimately, all these pathways lead to a process called RNA-induced silencing complex (RISC), in which the double-stranded miRNA is handed over to Argonaute, which in turn selects one strand (guide strand) to become the mature miRNA and discards the other strand (miRNA*). Finally, most mature miRNAs bind to the 3′untranslated region (3′UTR) of target messenger RNA (mRNA) to promote mRNA degradation and translational inhibition (O’Brien et al. 2018).

Although an overwhelming majority of miRNAs suppress the activity of their target mRNA, growing evidence reveals that some miRNAs upregulate gene expression by regulating miRNA-associated decay elements and blocking the function of repressive protein and thus activating other targets (Vasudevan 2012). Furthermore, several miRNAs appear to target the 5′UTR- rather than 3′UTR-binding site of mRNA (Ørom et al. 2008). To the best of our knowledge, the interaction between miRNA and 5′UTR-binding site has not been linked to the pathogenesis of ischaemic stroke.

## Involvement of miRNAs in the Pathophysiology of Ischaemic Stroke

### Energy Failure and Excitotoxicity

**Fig. 1:**
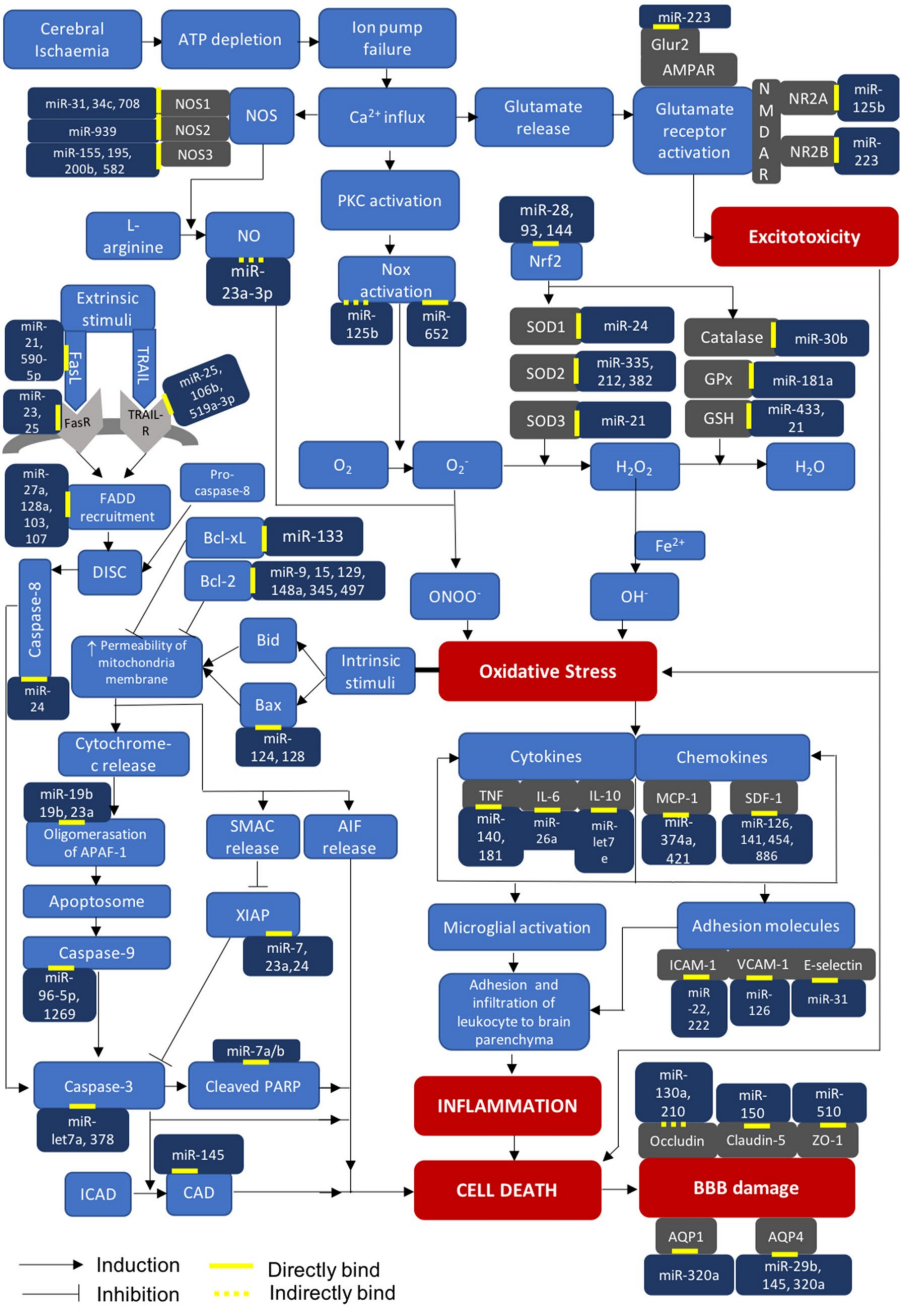
The involvement of key miRNAs and their targets in major mechanisms associated with ischaemic stroke. Abbreviations: *AIF* apoptosis-inducing factor; *AMPAR* α-amino-3-hydroxy-5-methyl-4-isoxazolepropionic acid receptor; *APAF-1* apoptotic protease-activating factor-1; *AQP-1* aquaporin-1; *AQP4* aquaporin-4; *Bcl-2* B-cell lymphoma-2; *Bcl-xL* B-cell lymphoma-extra large; *Ca*^*2*+^ calcium ions; *CAD* caspase-activated Dnase; *DISC* death-inducing signalling complex; *FADD* Fas-associated protein with death domain; *FasL* Fas ligand; *FasR* Fas receptor; *Fe*^*2*+^ ferrous ion; *Glur-2* glutamate receptor-2; *GPx* glutathione peroxidase; *GSH* glutathione; *H*_*2*_*O*_*2*_ hydrogen peroxide; *ICAD* inhibitor of caspase-activated Dnase; *ICAM-1* intercellular adhesion molecule-1; *IL-10* interleukin-10; *IL-6* interleukin-6; *MCP-1* monocyte chemoattractant protein-1; *miRNA* microRNA; *NMDAR* *N*-methyl-d-aspartate receptor; *NO* nitric oxide; *NOS* nitric oxide synthase; *Nox* nicotinamide adenine dinucleotide phosphate oxidase; *Nrf-2* nuclear factor erythroid-2; *O*_*2*_ oxygen; *O*_*2*_^*−*^ superoxide anion; *OH*^−^ hydroxyl radical; *ONOO*^*−*^ peroxynitrite; *PARP* poly(ADP-Ribose) polymerase; *PKC* protein kinase C; *SDF-1* stromal cell-derived factor-1; *SMAC* second mitochondria-derived activator of caspase; *SOD* superoxide dismutase; *TNF* tumour necrosis factor; *TRAIL* TNF-related apoptosis-inducing ligand; *TRAIL-R* TNF-related apoptosis-inducing ligand receptor; *VCAM-1* vascular cell adhesion molecule-1; *XIAP* X-linked inhibitor of apoptosis protein, *ZO-1* zonula occludens-1

The obstruction of cerebral blood flow during an ischaemic stroke significantly reduces the supply of oxygen and glucose to the brain tissue whereby hampers adequate generation of adenosine triphosphate (ATP). This in turn impairs the function of ion pumps, causes uncontrolled calcium depolarisation and triggers excessive production of excitotoxic neurotransmitters, notably glutamate and aspartate (Fig. 1). These then perturb the inner and outer mitochondrial membrane permeabilities and, as a result, lead to oxidative stress and induce cell death by augmenting the release of superoxide anion and cytochrome-*c*, respectively (Lai et al. 2014).

Increases in certain miRNAs, such as miR-107, closely followed by an increase in glutamate expression have been reported in both animal models and patients with ischaemic stroke (Yang et al. 2014). Decreased apoptosis and glutamate accumulation in neuronal cells exposed to ischaemia/reperfusion injury and in rats subjected to middle cerebral artery occlusion (MCAO) treated with miR-107 inhibitor corroborate the seminal role of miR-107 in the regulation of excitotoxicity during and after cerebral ischaemic injury. Luciferase assays aiming to identify the specific target genes for this particular miRNA have revealed that miR-107 reduces glutamate accumulation by directly binding to GLT-1, the most abundant glutamate transporter (Li et al. 2015; Yang et al. 2014). Similarly, overexpression of miR-223 has been shown to reduce neuronal excitability and neuronal cell death treated with a lethal dose of excitotoxic injury (500 μM of NMDA) as well as in mice subjected to a severe excitotoxicity (20 nmols of NMDA was directly injected into the striatum) or transient global ischemia. Suppression of glutamate receptor subunits Glur-2 and NR2B in the brain appears to account for the excitotoxicity regulatory effect of miR-223 (Harraz et al. 2012).

### Inflammation

Inflammation represents one of the major pathologies leading to neurovascular injury and hyperpermeability following an acute cerebral ischaemic injury and is mostly initiated by activation of various pro-inflammatory cytokines, chemokines and that of microglia, resident immune cells in the brain. Accumulating evidence indicates that, by targeting various genes, particular miRNAs play an important role in the development of post-ischaemic inflammatory responses (Abdullah et al. 2015; Wang et al. 2018). For instance, transfection of neuronal cells with miR-181c inhibits the expression of TNF, a prominent pro-inflammatory cytokine during the acute phase of an ischaemic injury and thus alleviates microglial activation and neuronal cell death. Direct targeting of TNF mRNA 3′UTR by miR-181c and subsequent decreases in TNF mRNA and protein expression have been shown to account for the above-mentioned benefits (Zhang et al. 2012). In addition to miR-181c, other miRNAs such as miR-216a, miR-3437b and miR-126-3p or -5p are also linked with the post-ischaemic cerebral levels of TNF. Hence, it was no surprise that attenuation of those particular miRNAs protected cerebrovasculature from ischaemic damage in animal models of stroke (Pan et al. 2020; Tian et al. 2018; Wang et al. 2018). Similar to these findings, the pharmacological inhibition or genetic deletion of miR-15a/16-1 activity has also been shown to reduce the expression of TNF alongside other pro-inflammatory cytokines, e.g. IL-6 and MCP-1 in a murine model of MCAO and improved neurobehavioral outcome as ascertained by rotarod, adhesive tape removal and foot fault tests at 3 days after MCAO (Yang et al. 2017a). In this context, modulation of miR-128 leading to suppression of p38α MAPK is also associated with decreases in pro-inflammatory cytokine release and ensuing inflammatory responses (Mao et al. 2017; Yang et al. 2017b).

As part of the post-ischaemic inflammatory process, the expression of adhesion molecules is upregulated in activated endothelial cells which facilitate adherence of leukocytes to them and paves the way for BBB disruption. Hence, mitigation of adhesion molecule expression by miRNAs following an ischaemic stroke may be of critical importance to protect neurovasculature (Ramiro et al. 2018). In support of this notion, administration of miR-126-3p/5p to mice subjected to MCAO has led to diminished BBB hyperpermeability, neurological impairments, leukocytes infiltration and the expression of pro-inflammatory mediators by directly regulating the levels of crucial adhesion molecules, VCAM-1 and E-selectin (Pan et al. 2020).

### Oxidative Stress

Oxidative stress is defined as a condition in which cells are subjected to excessive levels of molecular oxygen and its chemical derivatives called reactive oxygen species or ROS. Reperfusion following cerebral ischaemia evokes excessive generation of ROS which in turn induces cellular dysfunction by peroxidising lipids, denaturing proteins and altering intra- and intercellular signalling pathways. At tissue level, this dysfunction translates to enhanced platelet aggregation, vascular contractility, endothelial hyperpermeability and reduced cerebral blood flow (Allen and Bayraktutan 2009). Therefore, modulation of ROS generation during or after cerebral ischaemia by miRNA-mediated regulation of endogenous pro- and anti-oxidant enzymes may actually counterbalance these deleterious effects (Xu et al. 2018).

In accordance with this hypothesis, inhibition of miR-424 has been shown to protect neurovascular integrity by reducing the level of ROS and lipid peroxidation and concurrently increasing the expression of anti-oxidant MnSOD and extracellular SOD enzyme in mice with MCAO. Although this study did not perform luciferase assay to identify the target genes for miR-424, attenuation of these protective effects through knockdown of Nrf-2 (an important transcription factor that regulates endogenous anti-oxidant) and inhibition of SOD activity confirm the association between this particular miRNA and oxidative stress (Liu et al. 2015). In another study, administration of miR-93 to mice with MCAO has also led to marked increases in the expression of total SOD activity and promoted post-stroke recovery through directly binding to 3′UTR of Nrf-2 mRNA (Wang et al. 2016).

### Cell Death

All pathologies implicated in the pathogenesis of ischaemic stroke, namely energy failure, excitotoxicity, inflammation and oxidative stress can elicit regulated cell death in penumbra, the area surrounding the infarct, within hours to days of ischaemic injury and therefore exacerbate neurovascular damage (Rakkar and Bayraktutan 2016). Apoptosis, the classical and well-reported cell death form, can be activated through one of the two pathways. In the intrinsic pathway, the cell death occurs due to cell stress where the release of cytochrome-*c* from mitochondria plays a crucial role. In the extrinsic pathway, the cell death occurs because of signals from other cells where interaction between death receptor and its ligand on the cell surface plays a critical role.

An increase in the expression of pro-apoptotic genes makes up another important cause of cell stress in the intrinsic pathway (Chelluboina et al. 2014). This is partly regulated by the modulation of a number of miRNAs, including miR-214 and miR-128. Indeed, modulation of miR-214 following cerebral ischaemic injury reduces neuronal apoptosis by decreasing pro-apoptotic protein Bax and increasing anti-apoptotic protein Bcl-2 expression. While the downregulation of Bax may be due to the direct binding of miR-214 to this protein (Ping et al. 2017), the elevation of Bcl-2 level may be attributed to the inhibition of calcium overload. Namely, suppression of the mRNA-encoding sodium/calcium exchanger 1 (NCX1), a key regulator of calcium influx, along with various downstream effectors and pathologies that mediate cell death, notably oxidative stress, is thought to increase cell survival by upregulation of Bcl-2 expression and accounts for the protective role of miR-214 after ischaemic injuries (Aurora et al. 2012; Rakkar and Bayraktutan 2016). miR-128 has also been identified to directly bind to 3′UTR of Bax and thus increases cell survival (Adlakha and Saini 2011). Similarly, direct upregulation of a member of Bcl-2 anti-apoptotic protein family, i.e. Bcl-2l11 dramatically decreases neuronal apoptosis and improves post-stroke recovery of mice subjected to MCAO by suppressing miR-9 gene expression (Wei et al. 2016).

As indicated above, in the extrinsic pathway, the interaction between the cell surface death receptor (e.g. FasR) and its ligand (e.g. FasL) constitutes the main step in the initiation of events leading to apoptosis. By decreasing the expression of FasR, miRNAs like miR-25 reduce the number of apoptotic cells in ischaemic settings. Interestingly, by inhibiting the binding of FasR to its ligand, they also establish a balance between the expression of pro- and anti-apoptotic proteins and influence the intrinsic pathway as a result (Sprick and Walczak 2004; Zhang et al. 2016). Intrinsic and extrinsic pathways also interact at the point of caspase-3 activation. Caspase-3 is recognised as an executioner caspase in apoptosis due to its role in coordinating the destruction of cellular structures and macromolecules, notably cytoskeleton and DNA, respectively (Broughton et al. 2009; Shao and Bayraktutan 2014). Through direct targeting of caspase-3, a number of miRNAs such as miR-378 and miR-let-7c-5p have been shown to reduce the rates of cellular apoptosis following a cerebral ischaemic injury (Fang et al. 2012; Ni et al. 2015).

In addition to apoptosis, miRNA is also involved in the induction of other forms of ischaemic injury-mediated cell death such as autophagy, necroptosis and ferroptosis. Autophagy refers to the degradation of cytoplasmic proteins or organelles in order to produce vital amino acids to maintain cell survival in the absence of oxygen and glucose. While a degree of autophagy is thought to be protective for cellular homeostasis, excessive autophagy causes tissue damage and triggers functional aberrances (Zille et al. 2019). For instance, inhibition of miR-30a that binds to the mRNA of Beclin-1, the key molecule in autophagy, has been shown to prevent neuronal death and improve behavioural outcome of mice with ischemic stroke (Wang et al. 2014a). Necroptosis, on the other hand, is described as a type of regulated necrosis that is independent from caspase pathway and is characterised by the disruption of cell membrane and swelling of organelles (Datta et al. 2020). It is initiated by an interaction between cell death receptor (e.g. TNFR) and its ligand leading to activation of receptor-interacting serine/threonine-protein kinase-1 (RIPK1) and RIPK3. Once activated, RIPKs bind and phosphorylate mixed lineage kinase like protein (MLKL) whereby create MLKL oligomers which translocate to the plasma membrane and induce its permeabilisation (Vandenabeele et al. 2010). In this context, inhibition of miR-233-5p in an animal model of ischaemic stroke has been coupled to reduced neuronal death, infarct volume and neurological deficits through its regulatory effect on RIPK1/RIPK3/MLKL signalling pathway (Cuomo et al. 2019; Qin et al. 2016). Ferroptosis is a newly discovered form of regulated cell death triggered by the accumulation of lethal levels of iron-dependent lipid peroxides and is implicated in aggravation of neurovascular damage following a cerebral injury (Ratan 2020). Overexpression of miRNAs involved in ferroptosis, notably miR-212-5p, has recently been associated with improved learning and spatial memory in an animal model of traumatic brain injury by directly regulating prostaglandin-endoperoxide synthase-2 (Ptgs2), a marker of ferroptosis (Xiao et al. 2019).

### BBB Disruption

The unregulated opening of BBB during or after an ischaemic stroke causes the accumulation of circulating molecules within the brain parenchyma and thus induces formation of vasogenic oedema and haemorrhagic transformation, main causes of death within the first week of an ischaemic stroke. At molecular level, breakdown of BBB is characterised by an impairment of tight junctions (TJs) that are formed between adjoining brain microvascular endothelial cells through the interactions of crucial transmembrane proteins, claudin-5, occludin and zonula occludens-1 (ZO-1) (Gibson et al. 2014; Winkler et al. 2020). Through close regulation of these transmembrane proteins, various miRNAs have been shown to actively participate in ischaemic stroke-induced BBB leakage. For instance, overexpression of miR-150 appears to mitigate BBB hyperpermeability, infarct volume and neurological impairment in animal models of ischaemic stroke by regulating claudin-5 expression and endothelial cell survival (Fang et al. 2016). Again, increased expressions of occludin and ZO-1 have been shown to account for BBB-protective effects of miR-130a and miR-155, respectively in MCAO rats (Caballero-Garrido et al. 2015; Wang et al. 2017b).

Aquaporins (AQPs), the plasma membrane water-transporting proteins, also contribute to formation of BBB. Indeed, through direct regulation of AQPs, in particular, AQP4 which makes up the most abundant AQP in the central nervous system, exogenously administered miR-29b and miR-145 have been shown to preserve BBB function in mice subjected to MCAO (Wang et al. 2015; Zheng et al. 2017).

### Predictive Biomarker

Identifying generally healthy individuals who may be at risk for stroke is of critical importance to ameliorate the devastating impact of this condition globally. A large number of modifiable and non-modifiable risk factors such as hypertension, diabetes, atherosclerosis, age, sex and ethnicity are associated with an increased risk of stroke (Vijayan and Reddy 2016b). Inevitably, several miRNAs connected with the pathogenesis of these risk factors, including miR-155 (hypertension), miR-33 (hyperlipidaemia), miR-144 and miR-223 (diabetes mellitus) and miR-21, miR-126, and miR-320b (atherosclerosis) have been proposed as potential predictive markers for stroke (Rink and Khanna 2010; Vijayan and Reddy 2020). Given that Framingham stroke risk score (FSRS), a widely used risk score to predict 10-year probability of cerebrovascular events in asymptomatic individuals, tends to overestimate cerebrovascular events in general population, the new markers are desperately needed (Bineau et al. 2009; Dufouil et al. 2017; McClure et al. 2014). Exclusion of the functional status of endothelium may be the main reason for the above-mentioned overestimation of future strokes (Flueckiger et al. 2018; Liu and Wang 2016). Endothelial dysfunction is strongly associated with the future vascular diseases in asymptomatic adults. So, adding microvascular endothelial status to the FSRS may improve risk discrimination power (Flammer et al. 2012; Prugger et al. 2013; Zhong et al. 2018). Since several miRNAs have been identified as crucial regulators of endothelial function, it is possible that they can also, individually or collectively, serve as potential predictors of future strokes in general population.

In this regard, a large cohort study has reported that concurrent analysis of a 3-miRNA combination model, composed of miR-1268b, miR-4433b-3p and miR-6803-5p, exhibit a sensitivity of 80%, specificity of 82% and area under receiver curve (AUC) value of 0.89 to predict future stroke event in asymptomatic adults. As sensitivity and specificity quantify the percentages of true-positive subjects with disease and true-negative subjects without disease, they are regarded as reliable measures of predictive biomarkers or diagnostic tests. Since the results of sensitivity and specificity might vary according to the chosen cut-off point, the area under receiver-operating characteristic curve was also performed in this study to define the optimum cut-off points by plotting the sensitivity on the *y*-axis against specificity on the *x*-axis. Taken together, the observation of remarkably high sensitivity, specificity and AUC value in this study proposes the 3-miRNA combination model as an important predictor of future strokes. Although scrutiny of a large sample size (1612 participants with no history of stroke and 190 patients with previous stroke) substantiates the predictive value of these miRNAs, homogeneity of study population should not be dismissed while interpreting the results (Sonoda et al. 2019).

Epidemiological studies assessing the contribution of genetic variation to stroke risk report that there is substantial heritability for ischaemic stroke (37.9% for all ischaemic stroke), and this varies for different stroke subtypes; 16.1% for small-vessel disease, 32.6% for cardioembolic disease and 40.3% for large-vessel disease (Bevan et al. 2012)*.* In this context, increasing evidence shows that the specific single nucleotide polymorphisms (SNPs) in miRNA genes may affect the generation and function of mature miRNAs. Since SNPs are inherited genetic variations, they can be used to predict future cerebrovascular events. In a meta-analysis involving 3372 patients with stroke and 4394 controls, SNPs in miR-149 have been correlated with a significant increase risk of future stroke in East Asian population. This study clearly indicates that identification of genetic variation in the specific miRNA may be a potential strategy to predict risk of future strokes (Du et al. 2017).

### Diagnostic Biomarker

The accurate identification of stroke type is an important prerequisite for an immediate start of treatment. Owing to its widespread availability and speed, CT scan has become the most commonly used imaging procedure for the initial diagnosis of stroke type, haemorrhagic or ischaemic. MRI is another imaging modality that is occasionally used to diagnose strokes. Despite capturing more details compared to CT, MRI is a more expensive and relatively less available procedure. Besides, to avoid variability in image interpretation, it is recommended that CT is used as an initial imaging procedure (Parody et al. 2015; Wardlaw Joanna et al. 2007).

The panel of protein biomarkers such as activated protein C–protein C inhibitor complex (APC–PCI), glial fibrillary acidic protein (GFAP) and retinol binding protein 4 (RBP4) are potential alternative option to neuroimaging techniques, with advantages that can elucidate the underlying mechanism of disease, but they have shown limited diagnostic value with regard to blood-based biomarkers in stroke patients (Misra et al. 2017). Utilisation of circulating miRNAs as a blood-based biomarker in acute stroke patients has recently become an attractive and feasible concept (Table 1)*.* Unlike intracellular mRNAs, miRNAs exhibit remarkable stability and resistance to nuclease digestion as well as other harsh conditions such as boiling, low or high pH, extended storage and freeze–thaw cycles (Wang et al. 2013). In addition, analysis of circulatory levels of certain miRNAs can help monitor both cerebral and systemic changes after an ischaemic stroke (Eyileten et al. 2018). Comprehensive evaluation of circulating miRNAs by a gradual approach of discovery, validation and replication has pinpointed the combination of miR-125a-5p, miR-125b-5p and miR-143-3p as an important diagnostic biomarker for ischaemic stroke with remarkable sensitivity (85.6%), specificity (76.3%) and AUC value (0.90) (Tiedt et al. 2017). In comparison, multimodal cranial CT scan has a sensitivity of 72.5% for ischaemic stroke while commonly used high-sensitivity biomarkers like C-reactive protein (0.73 vs 0.90), IL-6 (0.82 vs 0.90) and neuron-specific enolase (0.69 vs 0.90) have significantly lower AUC values than these three miRNAs. Importantly, despite differentiating ischaemic stroke from transient ischaemic attack (TIA), the changes in these miRNA levels failed to correlate with infarct volume. Subsequent experiments proving platelets as the major cellular source of these miRNAs have explained why changes in these miRNA levels do not correlate with infarct volume or neuronal cell death. Similar studies have reported that the level of serum miR-221-3p and miR-382-5p may be an independent predictor of acute ischaemic stroke, with AUC values of 0.810 and 0.748, respectively (Tsai et al. 2013; Wang et al. 2017a).

**Table 1:** Observational studies investigating the diagnostic and prognostic role of miRNAs in patients with ischaemic stroke

Study design		Key findings	
Author (year)		Diagnostic marker	Prognostic marker
Zeng et al. (2011)	Case–control study	miR-210—an independent predictor of AIS, with AUC value, sensitivity and specificity of 0.65, 88.3% and 41.1%	miR-210—an independent predictor for poor outcome (mRS > 2), with AUC value, sensitivity and specificity of 0.64, 83.7% and 50.7%
	*N* = AIS patients (112) vs HCs (60)	No correlation between miR-210 level and stroke subtypes based on TOAST classification	
	Sample source: whole blood		
	Methodology: RT-PCR		
	Blood collection time: days 3, 7 and 14 after symptom onset		
Long et al. (2013)	Cross-sectional study	miR-30a–AUC: 0.91–0.93; sensitivity: 90–94%; specificity: 80–84%	Not evaluated
	*N* = AIS patients (197) vs. HCs (50)	miR-126–AUC: 0.92–0.94; sensitivity: 90–92%; specificity: 82–86%	
	Sample source: plasma	miR-let-7b–AUC: 0.91–0.93; sensitivity: 80–84%; specificity: 82–86%	
	Methodology: qRT-PCR		
	Blood collection time: 24 h–48 weeks after symptom onset		
Leung et al. (2014)	Case–control study	Plasma miR-124-3p levels significantly increased in HS patients in comparison to AIS patients in cases presented ≤ 6 h after stroke onset. In contrast, the level of miR-16 was markedly higher in HS patients only in cases presented at 6–24 h after onset	Plasma concentrations of miR-124-3p correlated positively with lesion volume of HS patients
	*N* = AIS patients (74) vs. HS patients (19) vs. HCs (23)	To discriminate HS and AIS:	Plasma levels of miR-124-3p and miR-16 were not associated with lesion volume in AIS patients
	Sample source: plasma	miR-124-3p–AUC value, sensitivity and specificity of 0.7, 68.4% and 71.2%	
	Methodology: qRT-PCR	miR-16–AUC value, sensitivity and specificity of 0.66, 94.7% and 35.1%	
	Blood collection time: < 24 h after symptom onset		
Wu et al. (2015)	Case–control study	The combination of miR-15a, miR-16 and miR-17-5p level was independent predictor of AIS with AUC value of 0.845. Sensitivity and specificity were not provided	There was a significant association between miR-16 levels and HDL and ApoA1 expression
	*N* = AIS patients (106) vs. HCs (120)		No correlation between miR-17-5p and any clinical characteristic
	Sample source: serum		
	Methodology: qRT-PCR		
	Blood collection time: not provided		
Kim et al. (2015)	Cross-sectional study	miR-17 plasma level was an independent predictor of AIS, with AUC value, sensitivity and specificity of 0.642, 37.4% and 89.2%	The level of miR-17 was not correlated with infarct volume and stroke severity, but was associated with future stroke recurrence
	*N* = AIS patients (83) vs. HCs (37)		
	Sample source: plasma		
	Methodology: qRT-PCR		
	Blood collection time: < 7 days after symptom onset		
Tian et al. (2016)	Case–control study	The level of miR-16 was an independent predictor of AIS with AUC value of 0.775, sensitivity 69.7% and specificity 87%	MiR-16 expression was significantly higher in the poor prognosis (mRS 3–6) group than in the good prognosis (mRS 0–2) group
	*N* = AIS patients (33) vs. HCs (23)	miR-16 AUC value, sensitivity and specificity in AIS patients reached 0.95, 100% and 91.3% in stroke derived from large artery atherosclerosis	
	Sample source: plasma		
	Methodology: microarray and qRT-PCR		
	Blood collection time: < 6 h after symptom onset		
Huang et al. (2016)	Case–control study	Higher levels of miR-let-7e-5p were associated with increased risk of AIS (adjusted OR, 1.89; 95% CI 1.61–2.21, *p* < 0.001)	The level of miR-let-7e-5p correlated with platelet dysfunction
	*N* = AIS patients (346) vs. HCs (346)	The addition of miR-let-7e-5p to stroke traditional risk factor increased AUC value from 0.74 to 0.82. Sensitivity and specificity were not available	
	Sample source: whole blood		
	Methodology: qRT-PCR		
	Blood collection time: > 24 h after symptom onset		
Tiedt et al. (2017)	Case–control study	The combination of miR-125a-5p, miR-125b-5p, miR-143-3p level was an independent predictor of AIS with AUC value, sensitivity and specificity of 0.90, 85.6% and 75.6%	There was no correlation between infarct volumes and levels of miR-125a-5p, miR-125b-5p and miR-143-3p
	Discovery stage: *N* = AIS patients (20) vs. HCs (20)	To differentiate AIS patients with TIA patients, this combinational miRNA displayed AUC value, sensitivity and specificity of 0.66, 89.2% and 27.1%	
	Validation stage: *N* = AIS patients (40) vs. HCs (40)		
	Replication: *N* = AIS patients (200) vs. HCs (100) vs. TIA (72)		
	Sample source: plasma		
	Methodology: RNA sequencing and qRT-PCR		
	Blood collection time: < 24 h after symptom onset		
Wang et al. (2017a, b)	Case–control study	The level of miR-221-3p and miR-382-5p were an independent predictor of AIS, with AUC values of 0.810 and 0.748, respectively. Sensitivity and specificity were not available	The level of miR-221-3p and miR-382-5p were not associated with NIHSS score and with abnormalities in laboratory findings. However, miR-4271 were positively correlated with level of blood glucose
	*N* = AIS patients (79) vs. HCs (39)		
	Sample source: serum		
	Methodology: qRT-PCR		
	Blood collection time: < 6 h after symptom onset		
Chen et al. (2018)	Case–control study	The level of miR-146b was an independent predictor of AIS, with AUC value of 0.776. Sensitivity and specificity were not available	Increased level of miR-146b positively correlated with infarct volume and NIHSS score on admission
	*N* = AIS patients (128) vs. HCs (102)		miR-146b level was associated with hs-CRP and IL-6 level
	Sample source: serum		
	Methodology: qRT-PCR		
	Blood collection time: < 24 h after symptom onset		
He et al. (2019a, b)	Prospective cohort study	Not evaluated	The increased levels of miR-125b-5p and miR-206 were associated with higher NIHSS scores and greater infarct volume
	*N* = 94 AIS patients		miR-125b-5p levels were an independent predictive marker for unfavourable outcome (mRS > 2) after thrombolysis, with AUC value, sensitivity and specificity of 0.735, 86.36% and 55.36%
	Source: plasma		
	Methodology: RT-PCR		
	Blood collection time: < 24 h after symptom onset		
Zheng et al. (2019)	Case–control study	Not evaluated	The increased levels of miR-21-5p, miR-206 and miR-3123 were associated with the risk of haemorrhagic transformation in patients with cardioembolic stroke with AUC value of 0.67, 0.68 and 0.66, respectively
	*N* = 58 AIS patients		
	Sample source: plasma		
	Methodology: qRT-PCR		
	Blood collection time: < 24 h after symptom onset		
Kalani et al. (2020)	Cohort study	25 extracellular miRNAs have been identified to be significantly altered following stroke injury and were able to discriminate between ischaemic and haemorrhagic stroke with AUC value of 0.813	Not evaluated
	*N* = IPH (19) vs SAH (17) AIS (21)		
	Sample source: plasma		
	Methodology: RNA sequencing		
	Blood collection time: < 24 h after symptom onset		

*AIS* acute ischaemic stroke, *ApoA1* apolipoprotein A1, *AUC* area under receiver-operating characteristic curve, *CI* confidence interval, *HCs* healthy controls, *HDL* high-density lipoprotein, *HS* haemorrhagic stroke, *hs-CRP* high-sensitivity C-reactive protein, *IL-6* interleukin-6, *IPH* intraparenchymal haemorrhage, *miRNA* microRNA, *mRS* modified rankin scale, *N* number of participants, *NIHSS* national institutes of health stroke scale, *OR* odds ratio, *qRT-PCR* real time quantitative polymerase chain reaction, *RNA* ribonucleic acid, *SAH* subarachnoid haemorrhage, *TIA* transient ischaemic attack, *TOAST* trial of org 10172 in acute stroke treatment

In addition to its ability to differentiate patients with ischaemic stroke and healthy subjects, miRNAs may also be used to distinguish ischaemic stroke from haemorrhagic stroke. For instance, in a study involving 97 stroke patients, miRNA-124-3p has shown sensitivity of 68.4% and specificity of 71.2%, and miRNA-16 exhibited sensitivity of 94.7% and specificity of 35.1% to differentiate ischaemic stroke and haemorrhagic stroke. However, as patients within 24 h of ischaemic stroke have been recruited for this study, the results may not be truly indicative of the acute stroke settings (Leung et al. 2014). Recently, variations observed in the level of 25 extracellular miRNAs, e.g. miR-30a-3p, miR-224-5p, miR-98-3p, miR-629-5p and miR-320a, after stroke have also been shown to reliably differentiate ischaemic strokes from haemorrhagic ones with an AUC value of 0.813. However, large cohort studies focusing on a small number of these extracellular miRNAs may better validate particular miRNAs as diagnostic markers for stroke (Kalani et al. 2020).

A recent integrated meta-analysis, bioinformatics and data mining study investigating biomarkers for ischaemic strokes specifically induced by inflammation or infection has demonstrated miR-320b and miR-320d as key miRNAs involved in this process. Indeed, these miRNAs have been implicated in the pathogenesis of ischaemic strokes evoked by atypical infections caused by helicobacter pylori, amoebae and legionella bacteria or inflammation caused by systemic lupus erythematosus and asthma. Taken together, data discussed so far indicate that different miRNAs may serve as diagnostic marker for ischaemic stroke stemming from different causes (Xie et al. 2020).

### Prognostic Biomarker

A good prognostic biomarker for stroke should be able to predict short- and long-term disease-related complications, identify patients who may benefit most from the acute interventions and monitor the efficacy of the applied treatment over a specified time interval. Due to the considerable stability of miRNAs in the circulation and their extensive regulatory roles in cellular and molecular events after a cerebral injury, numerous studies have proposed miRNAs as crucial potential prognostic biomarkers for patients with ischaemic stroke (Martinez and Peplow 2016). For instance, the circulating level of miR-128, associated with neuroinflammation and neuronal cell death, is positively correlated with infarct volume, NIH stroke scale (NIHSS) score at 7 days and modified Rankin Scale (mRS) score at 3 months after an ischaemic stroke (Liu et al. 2019). Similarly, the combination of miR-124-3p, miR-125b-5p and miR-192-5p is correlated with the extent of neurological deterioration in ischaemic stroke patients treated with rtPA or underwent mechanical thrombectomy, with an AUC value of 0.803, a sensitivity of 88% and a specificity of 65.22%. Considering the inclusion of a small sample size in this study, these findings need to be confirmed by other studies (He et al. 2019a).

Haemorrhagic transformation (HT), referring to a wide spectrum of ischaemia-related cerebral haemorrhage, is a frequent complication of ischaemic stroke, particularly in patients who have been given thrombolytic therapy. It constitutes one of the main reasons leading to elevations in mortality and morbidity following ischemic strokes. Several miRNAs that are inextricably intertwined with the expression of basement membrane-degrading enzyme MMP-9, notably miR-21-5p (AUC value of 0.677), miR-206 (AUC value of 0.687) and miR-3123 (AUC value of 0.661) appear to predict the risk of HT in patients with cardioembolic stroke (Abdullah and Bayraktutan 2016; Zheng et al. 2019).

It is known that individuals who suffer a first stroke are at considerably greater risk of having another stroke compared to general population. It is also well known that stroke survivors who suffer a second stroke are ~ 3 times more likely to die from than those who do not experience a second stroke (Jørgensen et al. 1997). Scrutiny of the correlation between the level of atherosclerosis-related miRNAs and the risk of stroke recurrence has identified miR-17 as an important miRNA during the first 24 months after a stroke. However, only 120 participants (83 acute ischemic stroke patients and 37 controls) from Korea were studied in this study. Hence, further studies with bigger sample size and heterogenic participants are required to establish the role of this particular miRNA in ischaemic stroke (Kim et al. 2015)

## Therapeutic Strategy

Given that the alterations in expression of specific miRNAs contribute to the pathophysiological mechanisms underlying ischaemic stroke, manipulation of their levels through miRNA agomir or antagomirs may be a potential therapeutic approach (Table 2). For example, restoration of circulating levels of miR-424 by its agomir helped reduce cerebral infarct and oedema volumes in a mouse model of transient ischaemic stroke by inhibiting neuronal cell death, microglia activation and oxidative injury (Liu et al. 2015; Zhao et al. 2013). Likewise, pre-treatment with miR-200c antagomir significantly reduced infarct volume and neurological impairment in mice subjected to MCAO by directly regulating the protein expression of Reelin, an essential extracellular matrix protein for neuronal survival. In this study, the neurological deficits were measured by a neurological grading scale in which scores of 0 and 4 were defined by no observable neurological deficit and inability to walk, respectively. Inhibition of miR-200c dramatically accelerated the speed of recovery from ischaemic stroke (Stary et al. 2015).

**Table 2:** Animal studies investigating the therapeutic efficacy of miRNA in the setting of ischaemic stroke

Agent (Author)	Design	Key findings
miR-27b antagomir	Model: MCAO mice	Increased neuronal survival and promoted neurogenesis by directly regulating AMPK expression
(Wang et al. 2019)	Delivery strategy: NA	Improved functional outcome and spatial memory
	Route: intravenous	
	Time of administration: day 7, 14 and 28 after MCAO	
miR-107 antagomir	Model: MCAO rats	Suppressed VEGF mRNA and protein expression and promoted angiogenesis through directly binding to Dicer-1
(Li et al.)	Delivery strategy: NA	Reduced infarct volume and improved capillaries in ischaemic boundary zone
	Route: intraventricular	
	Time of administration: 1 h after MCAO	
miR-126 agomir	Model: MCAO mice	Promoted vascular remodelling and neurogenesis
(Qu et al.)	Delivery strategy: lentiviral vector	Improved neurobehavioral recovery and reduced brain atrophy volume
	Route: intracerebral	
	Time of administration: 7 days after MCAO	
miR-126-Primed EPCs	Model: MCAO mice	Increased proliferation, migration and tubulogenic capacity of EPCs
(Pan et al.)	Delivery strategy: lentiviral vector	Decreased ROS and increased NO production of EPCs by activating PI3K/Akt/eNOS pathway
	Route: intravenous	miR-126 augmented the therapeutic efficacy of EPCs and helped attenuate infarct volume and neurological deficits while improving cerebral blood flow, microvascular density and angiogenesis
	Time of administration: 2 h after MCAO	
miR-126-3p or -5p agomir	Model: MCAO mouse	Reduced cerebral infarct and oedema volumes
(Pan et al.)	Delivery strategy: lentiviral vector	Improved zonula occludens-1 and occlusion expressions and maintained BBB function
	Route: stereotactic injection	Reduced pro-inflammatory cytokine (IL-1β and TNF) and adhesion molecule (VCAM-1 and E-selectin) expressions
	Time of administration: 2 weeks before MCAO	
miR-132 agomir	Model: MCAO mice	Suppressed MMP-9 expression and maintained tight junction VE-cadherin and β-catenin levels
(Zuo et al. 2019)	Route: intraventricular	Reduced infarct and oedema volumes, as well as neurological deficits
	Delivery strategy: NA	
	Time of administration: 2 h before MCAO	
miR-155 antagomir	Model: distal MCAO mouse	Improved blood flow and microvascular integrity and reduced neuronal damages in the peri-infarct area
(Caballero-Garrido et al.)	Delivery strategy: novel locked nucleic acid technology	Reduced infarct size and neurological impairments
	Route: intravenous	Maintained the integrity of tight junctions through improved zonula occluden 1 protein
	Time of administration: 48 h after MCAO	Prevented post-ischaemic inflammation by decreasing cytokine and chemokine gene expressions
miR-195 agomir	Model: MCAO rats	Decreased inflammatory response and neuronal cell death via direct suppression of NF-κB and Sema3A/Cdc42/JNK signalling pathways
(Cheng et al.)	Delivery strategy: lentiviral vector	Stimulated proliferation and mobilisation of neural stem cells toward infarct area
	Route: intravenous	Improved neurological function and reduced infarct size
	Time of administration: 6 h after MCAO	
miR-195 agomir	Model: MCAO rats	Reduced neuronal cell death by downregulating KLF-5 and JNK expressions
(Chang et al. 2020)	Delivery strategy: rAAV2/EGFP vector	Decreased infarct volume and neurological deficits
	Route: Intravenous	Promoted neuronal growth, axonal regeneration and synaptic remodelling
	Time of administration: Not available	
miR-200c antagomir	Model: MCAO mice	Increased neuronal survival rates through directly binding to Reelin
(Stary et al.)	Delivery strategy: NA	Reduced infarct volume and neurological deficits
	Route: intraventricular	
	Time of administration: 24 h before MCAO	
miR-214 agomir	Model: MCAO mice	Inhibited neuronal cell death by directly binding to Bax protein
(Ping et al.)	Delivery strategy: NA	Improved neurological outcomes and reduced infarct volume
	Route: intraventricular	
	Time of administration: 48 h before MCAO	
miR-216a agomir	Model: MCAO mice	Downregulated pro-inflammatory mediators, e.g. iNOS, MMP-9, TNF and IL-1β by directly targeting JAK2/STAT signalling pathway
(Tian et al.)	Delivery strategy: NA	Improved functional outcomes and reduced infarct volume
	Route: intraventricular	
	Time of administration: 10 min after MCAO	
miR-365 antagomir	Model: MCAO rats	miR-365 targets Pax6, a transcription factor account for conversion of astrocytes to neurons
(Mo et al. 2018)	Delivery strategy: NA	Promoted neurogenesis by inducing new mature neuron generation derived from astrocytes in the ischemic striatum
	Route: intraventricular	Reduced neurological deficits and infarct outcome
	Time of administration: 30 min after MCAO	
miR-384-5p agomir	Model: MCAO mouse	Promoted proliferation and angiogenesis of EPCs by regulating Notch signalling pathway
(Fan et al. 2020)	Delivery strategy: NA	Decreased infarct size and neuronal cell death
	Route: Intraventricular	
	Time of administration: 2 days before MCAO	
miR-1906 agomir	Model: MCAO mice	Reduced post-stroke inflammatory response by directly targeting TLR-4
(Xu et al. 2017)	Delivery strategy: NA	Decreased neurological deficits and infarct volume
	Route: intraventricular	
	Time of administration: not provided	
miR-3473b antagomir	Model: MCAO mouse	Prevented neuroinflammation by downregulating mRNA and protein expression of pro-inflammatory mediators like iNOS, COX-2, TNF and IL-6
(Wang et al. 2019)	Delivery strategy: NA	Reduced infarct volume and improved neurobehavioural recovery
	Route: intraventricular	
	Time of administration: 3 days prior to MCAO	

*AMPK* 5′ adenosine monophosphate-activated protein kinase, *Cdc42* cell division control protein 42, *COX-2* cyclooxygenase-2, *eNOS* endothelial nitric oxide synthase, *EPCs* endothelial progenitor cells, *IL-1β* interleukin-1β, *IL-6* interleuikin-6, *iNOS* inducible nitric oxide synthase, *JAK* janus kinase, *JNK* c-Jun N-terminal kinase, *KLF-5* kruppel-like factor, *MCAO* middle cerebral artery occlusion, *miRNA* microRNA, *MMP-9* matrix metallopeptidase-9, *mRNA* messenger ribonucleic acid, *NA* not applicable, *NF-κB* nuclear factor-κB, *NO* nitric oxide, *Pax6* paired box protein-6, *PI3K* phosphoinositide 3-kinases, *rAAV2/EGFP* recombinant adeno-associated virus vector/E-green fluorescent protein,* ROS* reactive oxygen species, *Sema3A* semaphorin 3A, *STAT* signal transducer and activator of transcription, *TLR-4* toll-like receptor-4, *TNF* tumour necrosis factor, *VCAM-1* vascular cell adhesion protein-1, *VEGF* vascular endothelial growth factor

Following cerebral ischaemia, neural progenitor cells (NPCs) proliferate and migrate towards the ischaemic area and differentiate into few other cell lines, including neurons, glia and oligodendrocytes, to promote post-stroke neurogenesis (Hoehn Benjamin et al. 2005). In this regard, inhibition of miR-17-92 cluster has been found to induce proliferation, differentiation and survival of NPCs in subventricular zone (SVZ) through direct targeting of phosphatase and tensin homologue. In accordance with this finding, treatments with miR-17-92 cluster antagomir have improved functional outcome and neuronal plasticity in the cerebral ischemic boundary zone of rats subjected to MCAO (Liu et al. 2013; Xin et al. 2017). Similar to NPCs, endothelial progenitor cells (EPCs), released from the bone marrow, also detect and repair endothelial damage through differentiating into mature endothelial cells or mediating the release of various growth factors in response to an ischaemic injury (Abdulkadir et al. 2020; Bayraktutan 2019). The number as well as proliferative, migratory and tube-forming capacity of circulating EPCs after cerebral damage are regulated by overexpression of miR-126. When used together with miR-126, EPCs were significantly more effective in reducing infarct volume and neurological deficits in a mouse model of ischaemic stroke (Pan et al. 2018). Along with generating endogenous stem cells to promote angiogenesis and neurogenesis, the treatment strategy after an ischaemic stroke should also aim to maintain the viability of resident cerebral endothelial cells. In this context, intraventricular administration of miR-107 agomir to a rodent model of permanent ischaemic stroke has led to increases in mRNA and protein expression of VEGF and, as a result, promoted migratory and tubulogenic capacity of resident cerebral endothelial cells and reduced overall infarct volume by improving number of capillaries in the ischaemic boundary zone (Zhu et al. 2015).

Intriguingly, no clinical trial has so far investigated the protective effect of miRNA-based approaches in stroke patients. A phase 2 multicentre randomised-clinical trial exploring the safety and feasibility of miRNA-based approaches has shown that administration of Miravirsen, a miR-122 antagomir to patients with chronic hepatitis C virus (HCV) genotype 1 infection effectively reduces HCV RNA levels without showing any dose-dependent toxicity (Janssen et al. 2013).

## Future Perspectives

Accumulating evidence indicates that changes in quantity of circulating miRNAs may serve as predictive, diagnostic or prognostic biomarkers for ischaemic stroke. Evidence also indicates that manipulation of certain miRNAs may be an efficacious therapeutic strategy to mitigate the burden of ischaemic stroke. Nevertheless, several issues need to be addressed before translating hitherto experimental findings into clinical settings. Due to variations in the source of miRNAs and in protocols used to detect, extract and isolate them, markedly different levels of miRNA and contradictory outcomes have been reported in different studies. For example, while circulating level of miR-223 was shown to negatively correlate with NIHSS score and infarct volume in an observational study involving 79 acute ischaemic stroke patients and 75 healthy subjects, in another study, the level of exosomal miR-223 was shown to positively correlate with NIHSS and mRS scores but not infarct volume (Chen et al. 2017; Wang et al. 2014b).

In addition, common protocols to detect circulating miRNAs, i.e. RT-PCR and microarray may have serious limitations to consistently detect changes in different miRNA expressions thereby necessitating exploration of alternative more reliable, faster and cheaper approaches (Ferraro et al. 2016; Vaca 2014). Isothermal PCR and ODG platform (optoi-destiNA genomics), an innovative platform that was established by the integration of a unique chemical-based method for nucleic acid detection with a novel silicon photomultiplier-based reader, has recently emerged as important diagnostic utilities for direct qualitative and quantitative measurement of miRNAs in biological samples (Detassis et al. 2019; Gines et al. 2020).

Moreover, the discrete patterns of miRNAs expression at different time points of cerebral ischaemic injury potentially transform the therapeutic approach from a single-point, single-drug stroke therapy to a multiple-time-point and multi-drug therapeutic approach (Cai et al. 2019). For instance, miR-126 exhibits a crucial role for angiogenesis and neurogenesis and its level is markedly decreased at day 7 following an ischaemic injury. Hence, administration of miR-126 agomir at this time point is correlated with improved in vascular remodelling, neurogenesis and neurobehavioural outcome of MCAO mice (Qu et al. 2019). miR-195, on the other hand, plays an important role in regulating inflammatory responses after an ischaemic attack and its level begins to decrease from 3 h to 3 days in patients with ischaemic stroke. Hence, it is no surprise that the administration of miR-195 agomir within 6 h after cerebral ischaemic injury has been found to reduce the inflammatory response, neurological deficits and infarct size in MCAO rats (Cheng et al. 2019; Guang et al. 2018).

Since most studies reporting on the potential predictive, diagnostic and prognostic role of miRNA recruited homogenous populations and analysed limited number of samples, further studies with heterogonous participants are required to ascertain the true value of miRNAs as biomarkers. Although therapeutic efficacy of miRNA agomirs or antagomirs has been established in experimental settings, the optimum route and time point for their delivery remain to be established. Given that the short therapeutic window for thrombolysis renders most patients ineligible to receive rtPA, it is crucial to study and validate effectiveness of different miRNAs beyond the acute phase of stroke (Hisham and Bayraktutan 2013). Several studies attempting to address this critical issue exist and are listed in Table 2.

As a route of medication administration, intravenous route represents one of the most preferred approaches in clinical practice due to its safety and its ease of use. Nonetheless, due to structural and functional properties of the BBB, most exogenous substances administered through this route cannot enter the central nervous system. Although, direct administration of medication into the brain (i.e. intraventricular) may overcome such hurdles, such invasive procedures are difficult and associated with significant adverse effects. Intranasal administration may also be an effective delivery strategy in that agents inhaled appear to cross the BBB with ease and improved functional outcomes in some animal models of disease like Alzheimer (Mai et al. 2019). Novel delivery strategies utilising viral vectors, e.g. adeno-associated virus, or non-viral vectors, e.g. liposomes and exosomes, may mitigate most risks associated with other routes. Furthermore, the miRNAs inserted into a viral or non-viral vector can be continually expressed, resulting in strong downregulation or upregulation of miRNA of interest in any disease setting including ischaemic stroke (Li et al. 2018)*.*

It is noteworthy here that an alternative approach to validate miRNA targets should be explored to better understand how individual miRNAs regulate their target gene expression. It is thought that most miRNA–mRNA interactions involve the seed region at the 5′ end of the miRNA. However, most seed sequences only have seven or eight nucleotides; thus, one mRNA may have multiple miRNA targets or one miRNA can bind to multiple mRNAs. Therefore, binding of miRNAs to unintended mRNA(s) may potentially cause adverse effects due to up- or down-regulation of nonspecific genes (Li et al. 2013).

Future clinical studies should also consider the fact that most of the currently available studies investigating the effects of miRNA have been performed using animal models of ischaemic stroke and the affinity of miRNA to its target gene may be different in humans (Sundermeier and Palczewski 2016).

## Conclusion

Discovery of an intricate involvement of various miRNAs in the pathogenesis of major events, such as excitotoxicity, oxidative stress and BBB damage, promoting ischaemic stroke has implied that some of these miRNAs may serve as important therapeutics or clinical biomarkers for stroke. Despite availability of evidence demonstrating that different miRNAs can serve as blood-based biomarkers for the prediction, diagnosis and prognosis of ischaemic stroke, further studies with larger sample size are required to ascertain their value and reliability in heterogenous populations. Again, available data indicate that treatments with various miRNA agomirs or antagomirs remarkably improve neurological outcome while preventing infarct and oedema expansion in animal models of ischaemic stroke through in part promoting the recruitment and differentiation of progenitor cells. Although evidence regarding the time of treatment, administrative routes and delivery strategy continue to accumulate, further evidence is needed to devise a standard protocol for the use of miRNA as therapeutics in clinical settings.

In conclusion, miRNAs possess great potential to serve as early predictive markers, point-of-care diagnostic tools, reliable prognostic markers and efficacious therapeutics for ischaemic stroke.
